# Electrostatic Spray Deposition-Based Manganese Oxide Films—From Pseudocapacitive Charge Storage Materials to Three-Dimensional Microelectrode Integrands

**DOI:** 10.3390/nano7080198

**Published:** 2017-07-26

**Authors:** Richa Agrawal, Ebenezer Adelowo, Amin Rabiei Baboukani, Michael Franc Villegas, Alexandra Henriques, Chunlei Wang

**Affiliations:** 1 Department of Mechanical and Materials Engineering, Florida International University, Miami, FL 33174, USA; ragra005@fiu.edu (R.A.); eadel004@fiu.edu (E.A.); arabi009@fiu.edu (A.R.B.); mvill224@fiu.edu (M.F.V.); ahenr041@fiu.edu (A.H.); 2 Advanced Materials Engineering Research Institute (AMERI), Florida International University, Miami, FL 33174, USA; 3 Center for the Study of Matter at Extreme Conditions (CeSMEC), Florida International University, Miami, FL 33199, USA

**Keywords:** pseudocapacitors, hausmannite Mn_3_O_4_, birnessite MnO_2_, electrostatic spray deposition, electrochemical activation, carbon microelectromechanical systems (C-MEMS), microsupercapacitors

## Abstract

In this study, porous manganese oxide (MnO*_x_*) thin films were synthesized via electrostatic spray deposition (ESD) and evaluated as pseudocapacitive electrode materials in neutral aqueous media. Very interestingly, the gravimetric specific capacitance of the ESD-based electrodes underwent a marked enhancement upon electrochemical cycling, from 72 F∙g^−1^ to 225 F∙g^−1^, with a concomitant improvement in kinetics and conductivity. The change in capacitance and resistivity is attributed to a partial electrochemical phase transformation from the spinel-type hausmannite Mn_3_O_4_ to the conducting layered birnessite MnO_2_. Furthermore, the films were able to retain 88.4% of the maximal capacitance after 1000 cycles. Upon verifying the viability of the manganese oxide films for pseudocapacitive applications, the thin films were integrated onto carbon micro-pillars created via carbon microelectromechanical systems (C-MEMS) for examining their application as potential microelectrode candidates. In a symmetric two-electrode cell setup, the MnO*_x_*/C-MEMS microelectrodes were able to deliver specific capacitances as high as 0.055 F∙cm^−2^ and stack capacitances as high as 7.4 F·cm^−3^, with maximal stack energy and power densities of 0.51 mWh·cm^−3^ and 28.3 mW·cm^−3^, respectively. The excellent areal capacitance of the MnO*_x_*-MEs is attributed to the pseudocapacitive MnO*_x_* as well as the three-dimensional architectural framework provided by the carbon micro-pillars.

## 1. Introduction

The technological advancement toward small-scale and portable devices has resulted in an increased demand for micro-power systems. For instance, the recent boom in the development of implantable medical devices, wireless sensors, smart cards, microelectromechanical systems (MEMS) and personal electronics has resulted in the need for reliable miniaturized energy storage devices. At present, the majority of the micro-devices rely on batteries to provide the required energy and power. Despite the commercial availability of thin-film or “microbatteries”, their relatively poor power-handling ability and limited lifetime hinder their applicability to systems that require high current spikes [1,2]. As an alternative to batteries, energy harvesters hold significant promise for sustainable environments; however, the currently existing energy harvester systems require an energy storage device in tandem [1]. Electrochemical capacitors or supercapacitors, on the other hand, are electrochemical energy storage systems that possess higher power densities than batteries along with superior lifetime. Conventional supercapacitors, however, are too bulky for small-scale applications and their fabrication methods are not compatible with the currently existing Integrated Circuit (IC) technology. Therefore, of immediate need is downsizing supercapacitors with compatible microelectronic fabrication techniques, so that they can be placed directly on the chip. Such devices, also referred to as micro-supercapacitors (MSCs), generally possess negligible active material masses and, therefore, their performance metrics are typically normalized by the area of the system. The typical areal energy and power densities delivered by MSCs range from µWh-mWh*∙*cm^−2^ and µW-mW*∙*cm^−2^ [1,3,4,5]. Volumetric/stack normalization is also popular for reporting MSC performance, since it provides insight into intrinsic material properties, as well as device architecture.

In the past decade, carbon microelectromechanical systems (C-MEMS) technique has emerged as a potential technique to successfully fabricate carbon-based current collectors and electrodes for on-chip energy storage and bio-sensing applications [6,7,8,9,10,11,12,13]. The C-MEMS process is a microfabrication technique that essentially involves the pyrolysis of a patterned photoresist into carbon structures. C-MEMS offers the feasibility of creating 3D micro-pillars, which offsets the limitation of the small footprint area required for miniaturized systems. The idea of using 3D C-MEMS-based microstructures as current collectors for the integration of other capacitive materials was first demonstrated by Chen et al. [11], where they grew carbon nanotubes (CNTs) on the surface of the C-MEMS structures. The CNT/C-MEMS structures were reported to exhibit specific capacitance as high as 20 times that of the bare C-MEMS structures. Other reports have documented the use of C-MEMS-based MSCs including electrochemically activated C-MEMS [12], and polypyrrole (PPY) decorated C-MEMS structures [13]. Apart from CNT and PPY, manganese oxides offer great promise as active materials, owing to their high theoretical specific capacitance, environmental benignity, large abundance, and low cost [14,15,16,17]. Of all polymorphs, birnessite MnO_2_, in particular, is very well suited for energy storage applications, given the simultaneous utilization of the double-layer capacitance as well as the Mn^3+^/Mn^4+^ redox couple [17]. Its layered structure exhibits edge-sharing MnO_6_ octahedra in the sheets, and the facile in-and-out cation motion from the interlayer region allows for partial electrolyte ion intercalation into the lamellar region [17]. One of the recently proposed synthetic routes to obtaining birnessite MnO_2_ (layered) is the electrochemical phase transition from hausmannite Mn_3_O_4_ (spinel) [18,19,20]. The synergy between the hausmannite and birnessite phases has been documented to yield superior current response as opposed to the pristine MnO_2_ or Mn_3_O_4_ phases [21,22]. Furthermore, as noted by Kim et al. [23], the spontaneous transition of layered to spinel manganese oxides is one of the critical factors that compromises with their structural stability, and therefore, the opposite transition from spinel to layered phases could be advantageous for enhancing the cycle life of layered birnessite materials. 

In this work, porous manganese oxide films were synthesized via electrostatic spray deposition (ESD) and evaluated as pseudocapacitive materials and as active materials for C-MEMS integration. ESD is an electrohydrodynamic spraying technique, which essentially involves the disintegration of a precursor solution into an aerosol spray upon the application of a high voltage between the feeding source and a grounded preheated substrate. The ability to tailor the film morphology by fine-tuning deposition parameters, without the need for vacuum, is what makes ESD an attractive and cost-effective thin-film synthesis method [24,25,26,27,28,29]. The ESD-derived manganese oxide films were able to deliver specific capacitances as high as 225 F∙g^−1^ from 72 F∙g^−1^ upon electrochemical cycling in neutral aqueous media. The enhancement in capacitance was ascribed to a partial phase transformation from hausmannite Mn_3_O_4_ (spinel) to birnessite MnO_2_ (layered). In addition to the enhancement in charge storage capacity, a concomitant improvement in kinetics and resistivity was observed upon cycling. Given the coexistence of the two phases, the films are referred to as MnO*_x_* henceforth. Several reports have documented the use of ESD-based manganese oxide films but their direct applicability to microsystems has not been explored [30,31]. Given the potent advantages of thin films for microsystems, the ESD-based MnO*_x_* were integrated onto the C-MEMS-generated 3D carbon micro-pillars and evaluated as MSC systems. In a two-electrode configuration, the MnO*_x_*/C-MEMS microelectrodes (MEs) were able to deliver specific capacitance as high as 0.055 F∙cm^−2^, much higher than other microsystems [32–34]; the maximal volumetric energy and power densities delivered by the MnO*_x_*/MEs were 0.51 mWh·cm^−3^ (1.84 J∙cm^−3^) and 28 mW*∙*cm^−3^ (28.3 mJ*∙*s^−1^*∙*cm^−3^), respectively. The excellent areal capacitance and the relatively high stack energy density of the MnO*_x_*-MEs are attributed to the pseudocapacitive MnO*_x_* as well as the three-dimensional architectural framework provided by the micro-pillars. 

## 2. Results and Discussion

### 2.1. Crystallographic, Spectroscopic and Microstructural Studies on the As-Prepared and Cycled MnO_x_ Films

**Figure 1:**
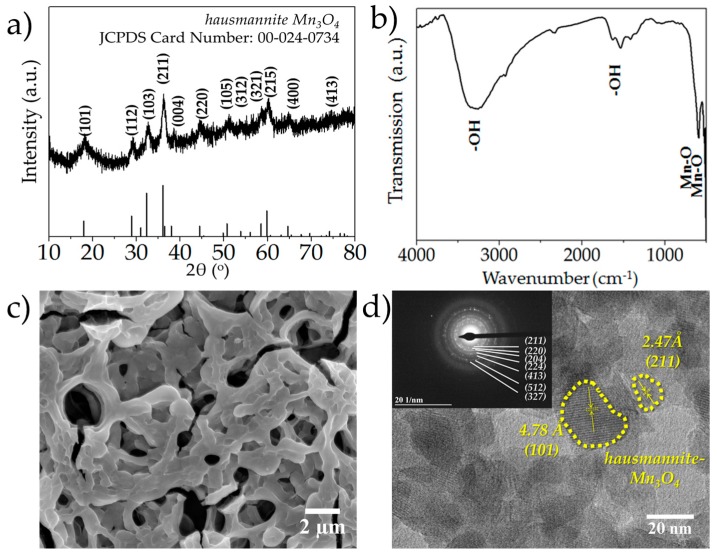
(**a**) XRD pattern of the as-deposited MnO*_x_* powders; (**b**) FTIR pattern of the as-deposited MnO*_x_* films; (**c**) SEM microstructure of the as-deposited MnO*_x_* films; (**d**) HRTEM micrograph of the as-deposited MnO*_x_* powders; the inset depicts the SAED pattern.

**Figure 2:**
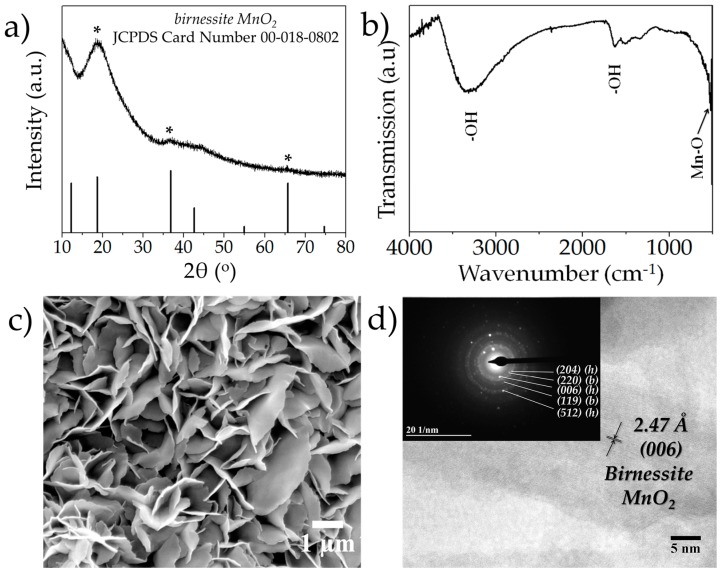
(**a**) XRD pattern of the cycled MnO_x_ powders; (**b**) FTIR pattern of the cycled MnO*_x_* films; (**c**) microstructure of the cycled MnO*_x_* films showing layered platelet-like morphology; (**d**) HRTEM of the cycled MnO*_x_* powders; the inset shows the SAED pattern, (**b**) represents birnessite phases, whereas (**d**) represents hausmannite phase.

The XRD pattern of the as-deposited manganese oxide powders is depicted in Figure 1a. The as-deposited powder was confirmed to be hausmannite-Mn_3_O_4_ (Joint Committee on Powder Diffraction Standards (JCPDS) Card Number: 00-24-0734). The peaks at 18.0°, 28.9°, 32.4°, 36.2°, 38.3°, 44.5°, 50.9°, 53.6°, 58.5°, 60.1°, 64.8° and 74.3° are attributed to the (hkl) planes (101), (112), (103), (211), (004), (220), (105), (321), (215), (400) and (413), respectively. The XRD pattern of the cycled MnO*_x_* (after 1000 cycles), depicted in Figure 2a, on the other hand, did not exhibit any sharp peaks indicating the phase to be mostly amorphous. The broad peak centered at 18.64° is indexed as (002) plane of the birnessite MnO_2_ phase, whereas the two faint peaks located at 36.7° and 65.7° are identified as (006) and (119) planes, respectively (JCPDS Card Number: 00-018-0802) [35]. The Fourier Transform Infrared (FTIR) spectrum of the as-synthesized MnO*_x_* films between frequencies of 500–4000 cm^−1^ is depicted in Figure 1b. In the high-frequency region, the broad peak centered at 3345 cm^−1^ is assigned to –OH stretching vibrations [36], whereas the peak located at around 1624 cm^−1^ is attributed to –OH bending vibrations [36]. In the lower frequency region, the absorption peaks located at 529 cm^−1^ and 602 cm^−1^ are ascribed to Mn-O stretching modes in the octahedral and tetrahedral sites, respectively [20,36,37,38]. The FTIR spectrum of the cycled MnO*_x_* (after 1000 cycles) film is shown in Figure 2b. It should be noted that while the majority of the peaks signaling –OH bending and stretching are still visible, the peak at 602 cm^−1^ which signals the presence of the tetrahedral stretch of Mn-O disappears, indicating a loss of order in the crystal structure as compared to the as-synthesized MnO*_x_* films. However, the peak at 525 cm^−1^ is still present indicating that the cycled films comprise predominantly Mn-O groups from the octahedral layers, which is also indicative of birnessite phase. The microstructure of the as-deposited manganese oxide sample is shown in Figure 1c; the microstructure was mostly porous with reticular network-like morphology. The microstructure of the electrochemically cycled manganese oxide (after 1000 cycles) is shown in Figure 2c. As evident, there is a dramatic change in the morphology as compared to the as-deposited films; as opposed to the previous reticular structure, the post-cycling structure is predominantly “layered” platelet-like, which is reminiscent of birnessitic-MnO_2_ [19,20]. Figure 1d shows the High Resolution Transmission Electron Microscope (HRTEM) micrograph of the sample; the particles were nano-sized with different planar orientations. The lattice fringes with d-spacings of 4.78 Å and 2.47 Å were indexed as (hkl) orientations of (101) and (211) of hausmannite-Mn_3_O_4_. The Selected Area Electron Diffraction (SAED) pattern is shown as the inset of Figure 1d. The d-spacings of 2.41 Å, 2.03 Å, 1.83 Å, 1.54 Å, 1.27 Å, 1.10 Å and 1.03 Å corresponded to (hkl) orientations of (211), (220), (204), (224), (413), (512) and (327) of hausmannite-Mn_3_O_4_. The HRTEM of the cycled MnO*_x_* powders is shown in Figure 2d and the detailed SAED analysis is shown as the inset. The d-spacings of 2.29 Å and 1.50 Å, correspond to (hkl) planes of (006) and (119) of the birnessite phase, respectively, whereas the d-spacings of 2.01 Å, 1.82 Å and 1.13 Å correspond to (220), (204) and (512) planes of the hausmannite phase.

### 2.2. Electrochemical Characterization of the MnO_x_ Films

**Figure 3:**
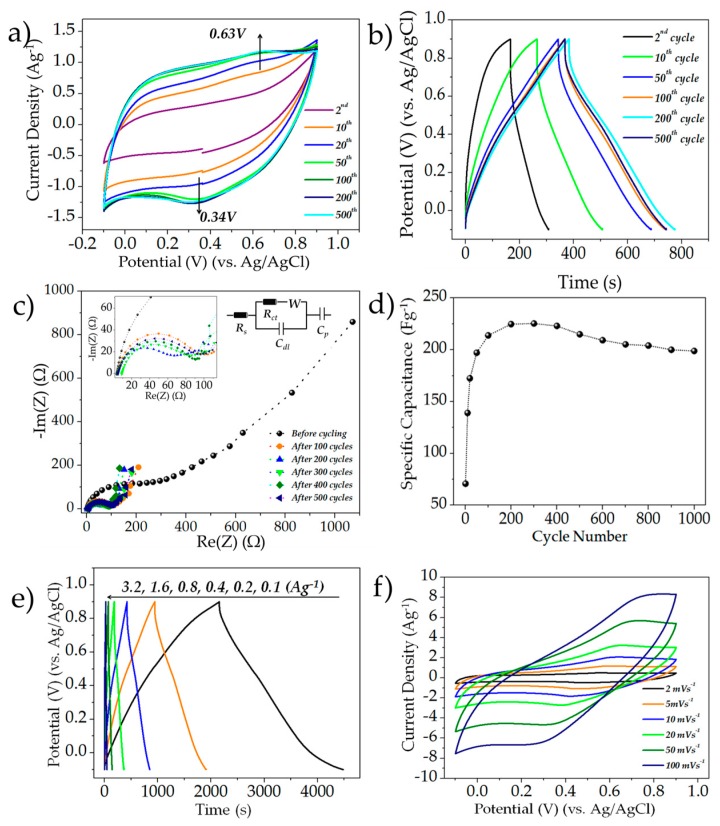
(**a**) CV curves of the MnO*_x_* films at different cycles scanned at a sweep rate of 5 mV∙s^−1^; (**b**) typical GCD curves of the MnO*_x_* films at a constant charge-discharge current rate of 0.5 Ag^−1^ for different cycles; (**c**) Nyquist plots of the MnO*_x_* films at different cycles; the inset depicts the equivalent circuit used for system analyses; (**d**) cycling behavior of the MnO*_x_* films; (**e**) galvanostatic charge-discharge profiles of the MnO*_x_* films at different current rates; (**f**) CV curves at different sweep rates (2–100 mV∙s^−1^).

The cyclic voltammograms (CV) of the as-synthesized manganese oxide films between potentials of −0.1 V and 0.9 V (vs. Ag/AgCl) at a sweep rate of 5 mV∙s^−1^ are shown in Figure 3a. As evident for the 2nd cycle, the CV has no discernible redox peaks. Upon cycling, however, there is a gradual origin and increase in the intensity of anodic and cathodic currents around 0.63 V and 0.34 V, which is attributed to the oxidation and reduction between the Mn^3+^/Mn^4+^ redox couple, as per the Pourbaix diagram [21,30,39]. It is worth noting that the area under the CV curve increases substantially upon cycling, which implies that there is enhancement in capacitance upon electrochemical cycling. The gravimetric specific capacitances (*C_s_*) at the 2nd, 10th, 20th, 50th, 100th, 200th and the 500th cycles were approximated as 56, 102, 129, 156, 162, 163 and 160 F∙g^−1^, respectively, using Equation (1), where *C_s_* is the specific capacitance, m is the mass of the electrode, *s* is the scan rate, *ΔV* is the voltage window, *I* is the current, and *V* is the voltage.

(1) $$C_{s}=\;\frac{1}{2ms\Delta V}\int IdV$$

Figure 3b depicts the galvanostatic charge-discharge (GCD) curves of the MnO*_x_* films at a current density of 0.5 Ag^−1^. As evident, there is a significant enhancement in capacitance from the 2nd cycle accompanied with a marked decrease in the voltage drop in the subsequent cycles. At the 2nd, 10th, 20th, 50th, 100th, 200th and 500th cycles, the specific capacitance was approximated as 72, 139, 172, 197, 214, 225, 223 and 215 F∙g^−1^, respectively. In order to investigate the resistivity changes upon subsequent cycling in the sample, Electrochemical Impedance Spectroscopy (EIS) studies were carried between frequencies of 100,000 Hz and 0.01 Hz. A typical Nyquist curve comprised a depressed semicircle in the high-frequency region followed by a relatively linear slope in the lower frequencies. The diameter of the semicircular region is associated with the charge-transfer resistance of the system at the electrode-electrolyte interface, and equivalent circuit analyses were done in order to verify the effect of cycling on the resistance of the system. The equivalent circuit for the Nyquist plots has been depicted as the inset of Figure 3c. *R_s_*, *R_ct_*, *W*, *C_dl_* and *C_p_* stand for solution resistance, charge-transfer resistance, Warburg impedance, double-layer capacitance, and pseudocapacitance, respectively [40]. The values of *R_ct_* and *R_s_* at different cycles have been tabulated in Table 1. 

As evident, the charge-transfer resistance decreases rapidly for the first 200 cycles, which can be ascribed to the transformation of the relatively insulating Mn_3_O_4_ to the more conducting birnessite MnO_2,_ and is consistent with previous reports [19]. The slight increase in the resistance after the 200th cycle can be attributed to the resistance changes in the predominantly active birnessite phase upon cycling. The long-term cycling of the MnO*_x_* films has been shown in Figure 3d. The specific capacitance of the electrode steadily increases from ca. 72 F∙g^−1^ at the second cycle to a maximal capacitance of 225 F∙g^−1^ at the 200th cycle. It should be noted that even the starting capacitance of the MnO*_x_* electrodes was superior to the previously ESD-synthesized β-MnO_2_ films, which yielded a low gravimetric capacitance of 13 F∙g^−1^ [41]. At the 1000th cycle, the capacitance dropped to 199 F∙g^−1^, resulting in a capacitive drop of approximately 11.6% when compared with the maximal capacitance reached by the system. The GCD curves at different current densities are shown in Figure 3e. The electrodes were able to deliver capacitances of 233, 221, 196, 167, 131 and 88 F∙g^−1^ at current densities of 0.1, 0.2, 0.4, 0.8, 1.6 and 3.2 Ag^−1^, respectively. The effect of scan rate on the capacitance of the MnO*_x_* films is shown in Figure 3f. The capacitances were estimated to be ca. 164, 136, 110, 77, 47 and 30 F∙g^−1^ at scan rates of 2, 5, 10, 20, 50 and 100 mV∙s^−1^, respectively. As evident, the capacitance of the MnO*_x_* electrodes decreases with increasing scan rate in addition to the clear deviation from the relatively rectangular capacitive shape. This is expected since the rapid electrolyte ion flux at higher scan rates limits the diffusion-controlled charge storage processes at the MnO*_x_* electrode surface, resulting in lower utilization of active charge storage sites, thereby limiting the charge storage [42]. 

### 2.3. MnO_x_/C-MEMS Characterization

**Figure 4:**
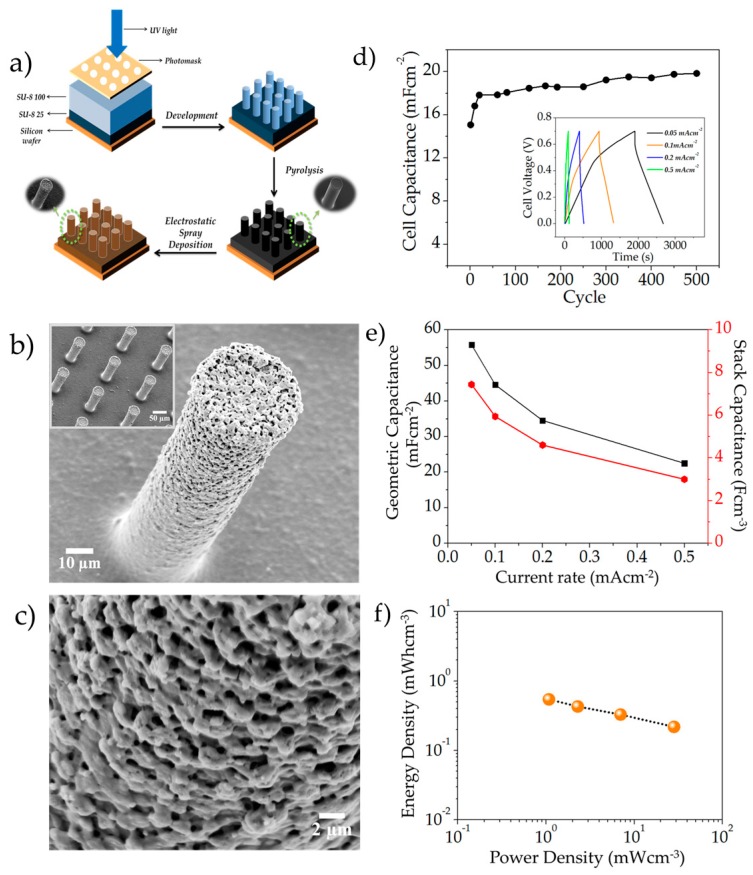
(**a**) Schematic representation of MnO*_x_*-ME fabrication process (detailed description in Section 3.3); (**b**) SEM micrograph of a typical MnO*_x_*-encrusted carbon micro-pillar; the inset shows MnO*_x_*-encrusted micro-pillar arrays; (**c**) zoomed-in view of the microstructure of the electrostatic spray deposition (ESD)-generated MnO*_x_* films, (**d**) cyclability of the MnO*_x_*-ME electrodes; the inset depicts typical charge–discharge curves of the MnO*_x_*-ME at different current rates (0.05–0.5 mA∙cm^−2^); (**e**) geometric and stack capacitances of the MnO*_x_*-MEs at different rates; (**f**) Ragone chart of the MnO*_x_*-MEs.

Figure 4a shows the typical fabrication process of the MnO*_x_*-MEs and the detailed explanation is given in Section 3.3. The typical MnO*_x_*-ME architecture is shown in Figure 4b. A typical micro-pillar had an average height of ~75 µm and a width of ~35 µm (adjusted for tilt angle). Figure 4c shows the magnified view of the side of the manganese oxide-encrusted micro-pillars; as evident, the films were reticular with very porous and consistent network-like morphology, analogous to the ones synthesized previously. The thickness of the manganese oxide film was approximately 0.6–0.8 μm. For stack energy and power density calculations, the energy and power were normalized with the volume of the electrodes, for which the height of the posts was multiplied with the footprint of the device (1 cm^2^), equating to a volume of *v* = 1 cm^2^ × 0.0075 cm = 0.0075 cm^3^. In order to evaluate the electrochemical performance of the MnO*_x_*-MEs, two identical MnO*_x_*-MEs were used in a two-electrode symmetric cell setup and the cell was analyzed between a voltage window of 0 and 0.7 V in 1 M Na_2_SO_4_. For the symmetrical cell system, one of the MnO*_x_*-MEs functioned as the working electrode, whereas the other served as the counter electrode. The typical cycling behavior of the MnO*_x_*-MEs is shown in Figure 4d. As evident, the cell capacitance increased from ≈14.7 mF∙cm^−2^ to ≈19.8 mF∙cm^−2^ from the 2nd to the 500th cycle, respectively. The enhancement in capacitance can be attributed to the electrochemical activation of the MnO*_x_* films upon cycling. GCD curves at different current densities are shown as the inset; the predominantly triangular shape of the charge-discharge curves indicates the capacitive nature of the microelectrodes. Figure 4e shows rate-handling capability of the system, the system was able to deliver geometric capacitances (normalized by footprint area) as high as 55 mF∙cm^−2^ at a current rate of 0.05 mA∙cm^−2^, while still maintaining a capacitance of 22.5 mF∙cm^−2^ at a current density of 0.5 mA∙cm^−2^. The maximal stack capacitance (normalized by volume) achieved was 7.44 F∙cm^−3^, resulting in a maximal stack energy density of 0.51 mWh∙cm^−3^ (1.84 J∙cm^−3^), whereas the maximal power density achievable was approximated as 28.3 mW∙cm^−3^ (28.3 mJ∙s^−1^∙cm^−3^), as shown in the Ragone chart in Figure 4f. The range of energy density achievable by the system was 0.21–0.51 mWh∙cm^−3^, for a power density range of 28.3–1.1 mW∙cm^−3^. It should be noted that the areal capacitance delivered by the MnO*_x_*-MEs was fairly high as compared to other microsystems reported in the literature [32,33,34], which is ascribed to the three-dimensional carbon micro-pillar framework which provides for a much larger surface area for active material integration. Despite the excellent areal capacitance and the relatively high energy density of the MnO*_x_*-MEs, their low power density needs to be addressed. Enhancement in the power handling is expected with the use of hybrid MnO*_x_* structures containing a combination of pseudocapacitive MnO*_x_* and conducting double-layer nanostructured carbons, as well as constructing on-chip inter-digitated systems. Designing inter-digitated designs with anode and cathode on the same chip reduces ion-transport resistance [1], as a result of which, the kinetics of the system can be enhanced. Devising such hybrid structures/inter-digitated systems to further enhance the power of MnO*_x_*-MEs is a subject of future works. 

## 3. Materials and Methods

### 3.1. Manganese Oxide Electrode Synthesis

Manganese (II) acetate tetrahydrate (Mn(CH_3_COO)_2_·4H_2_O, Alfa Aesar, Ward Hill, MA, USA) was first dissolved in 1,2-propanediol (Sigma Aldrich, St. Louis, MO, USA) in the ratio 2.4 mg·m∙L^−1^ with constant stirring for 30 min and directly used as the precursor solution in order to deposit the MnO*_x_* films. For the ESD setup, the feeding rate was kept at 3 mL·h^−1^ and the voltage between the needle and substrate was kept between 6 and 8 kV. The distance between the needle and the substrate was approximately 4 cm and the deposition was carried out at 300 °C on stainless steel substrates. The typical gravimetric mass yield was 1 ± 0.05 mg per sample. 

### 3.2. Structural and Material Characterization

Spectroscopic studies were carried out using FTIR spectroscopy in order to study the effect of cycling on the surface chemistry of the MnO*_x_* films using a JASCO FTIR 4100 equipped with an Attenuated Total Reflectance (ATR) accessory. For crystallinity studies, the powders were scratched off from the stainless steel substrates before and after cycling. The crystallinity of the as-deposited and cycled manganese oxide films was studied using a Siemens 5000D X-ray Diffractometer with Cu Kα radiation (Siemens, Munich, Germany). Further crystallographic studies on the as-deposited and cycled powders were carried out using a Philips CM-200 200 KeV Transmission Electron Microscope (TEM) (FEI Philips, Hillsborough, OR, USA). The morphology of the as-deposited and cycled films, as well as the MnO*_x_*/C-MEMS microelectrodes, was studied using a scanning electron microscope (JEOL SEM 6330F, Peabody, MA, USA) in the secondary electron imaging (SEI) mode. 

### 3.3. MnO_x_/C-MEMS Fabrication

The experimental setup and details of the C-MEMS process used in this work have been reported previously [3,4,5,6]. A schematic illustration of MnO*_x_*-ME fabrication is shown in Figure 4a. In brief, the C-MEMS-based 3D micro-pillars were prepared by a two-step photolithography process followed by a pyrolysis step. In the first photolithography step, a two-dimensional square (10 mm side) pattern was created using NANO^TM^ SU-8 25 (Microchem, Westborough, MA, USA), as the current collector. The photoresist film was spun-coated onto a silicon oxide wafer (4”, (1 0 0)-oriented, n-type) at 500 rpm for 12 s and at 3000 rpm for 30 s by using a Headway Research photoresist spinner, followed by soft baking at 65 °C for 3 min and hard baking at 95 °C for 7 min on a leveled hotplate. The baked photoresist was thereafter patterned with a UV exposure dose of 300 mJ·cm^−2^ using an OAI (800) Mask Aligner. After the exposure process, a post-expose bake was conducted at 65 °C for 1 min and 95 °C for 5 min on a hotplate. The second photolithography step comprised building the cylindrical micro-pillar arrays using NANO^TM^ SU-8 100 (Microchem, Westborough, MA, USA) on the patterned current collector. SU-8 100 was spun-coated at 500 rpm for 12 s and at 1500 rpm for 30 s using a Headway Research photoresist spinner. The spun-coated photoresist was then soft baked at 65 °C for 10 min on a leveled hot plate and hard baked at 95 °C for 45 min in an oven. The exposure was done using a UV exposure dose of 700 mJ·cm^−2^ using an OAI (800) Mask Aligner, following which a post-expose bake was performed at 65 °C for 3 min and 95 °C for 10 min in an oven. Afterward, the sample was developed by NANO^TM^ SU-8 developer (Microchem, Westborough, MA, USA) for 15–20 min to wash away any remaining unexposed photoresist, followed by isopropanol rinsing and nitrogen drying. Finally, the resultant SU-8 structures were pyrolyzed at 900 °C for 1 h in a Lindberg alumina-tube furnace with a continuous flow of argon at a ramp of 5 °C/min. After the carbonization process, the carbon samples were allowed to cool to room temperature naturally and directly used as substrates for MnO*_x_* integration using ESD as described in Section 3.1.

### 3.4. Electrochemical Characterization

The electrochemical characterization on the manganese oxide films deposited on stainless steel substrates as well as carbon micro-pillars was carried out using a Bio-logic Versatile Multichannel Potentiostat (VMP3). For half-cell studies, the MnO*_x_* films were used as the working electrode, a platinum wire served as the counter electrode, while an Ag/AgCl electrode served as the reference electrode; the MnO*_x_* films were tested between potentials of −0.1 V and 0.9 V (vs. Ag/AgCl). A neutral aqueous electrolyte of 1.0 M Na_2_SO_4_ was used for the cell assembly. For the MnO*_x_*/C-MEMS characterization, two of the manganese oxide-encrusted carbon micro-pillars were used in a symmetric configuration in 1.0 M Na_2_SO_4_ aqueous electrolyte for a cell potential of 0–0.7 V.

## 4. Conclusions

In this paper, manganese oxide films were synthesized using electrostatic spray deposition (ESD) and characterized as pseudocapacitive materials for electrochemical capacitor applications in neutral aqueous media. The initial phase synthesized was the relatively insulating hausmannite-Mn_3_O_4_, which partially transformed into the conducting birnessite-MnO_2_ upon electrochemical cycling, resulting in an enhanced gravimetric capacitance from 72 F∙g^−1^ to 225 F∙g^−1^. Furthermore, MnO*_x_*-MEs were created by combining bottom-up ESD approach and top-down C-MEMS approach. In a two-electrode setup, the MnO*_x_*-MEs were able to deliver geometric specific capacitances as high as 0.055 F∙cm^−2^, and maximal volumetric energy and power densities of 0.51 mWh∙cm^−3^ and 28.3 mW∙cm^−3^, respectively. The excellent areal capacitance and the high stack energy density of the MnO*_x_*-MEs are attributed to the pseudocapacitive MnO*_x_* as well as the three-dimensional architectural framework of the micro-pillars. The feasibility of using ESD for high gravimetric thin-film manganese oxide films and high areal capacitance 3D microelectrodes was therefore established. 

## Figures and Tables

**Table 1:** Solution and charge-transfer resistances computed from equivalent circuit at different cycles.

	Before Cycling	After 100 Cycles	After 200 Cycles	After 300 Cycles	After 400 Cycles	After 500 Cycles
***R_s_* (Ω)**	3.56	4.51	4.51	9.54	4.44	5.11
***R_ct_* (Ω)**	198.91	81.63	55.31	62.95	67.84	72
